# The effect of erector spinae plane block on postoperative extubation time and laboratory parameters in open heart surgery: a retrospective study

**DOI:** 10.55730/1300-0144.5772

**Published:** 2023-12-18

**Authors:** Feyza ÇALIŞIR, Yavuz ORAK, Ömer Faruk BORAN, Metin EGE, Mehmet Emin KARABEKİROĞLU, Hilal BİRADLI, Adem DOĞANER

**Affiliations:** 1 Department of Anesthesiology and Reanimation, Faculty of Medicine, Kahramanmaraş Sütçü İmam University, Kahramanmaraş, Turkiye; 2 Department of Biostatistics, Faculty of Medicine, Kahramanmaraş Sütçü İmam University, Kahramanmaraş, Turkiye

**Keywords:** Early extubation, early recovery, erector spinae plane block, coronary artery bypass surgery, pain management, troponin

## Abstract

**Background/aim:**

In open heart surgery, sternotomy causes inflammation in tissues, and inflammation causes postoperative pain. This study aims to examine the effects of bilateral erector spinae plane (ESP) blocks on postoperative extubation time and laboratory parameters in open heart surgery.

**Materials and methods:**

The study was managed using retrospective data from 85 patients who underwent open-heart surgery. Patients who received intravenous analgesia and were transferred to the intensive care unit with intubation were included in the study. Two groups were formed: those who received preoperative bilateral ESP block (ESB) and those nonblock (NB). Statistical significance was investigated between ESB and NB in terms of extubation time and laboratory parameters.

**Results:**

The postoperative extubation time for group NB was significantly longer at 360 (300–420) min compared to the observed 270 (240–390) min for ESB (p: 0.006). The length of stay in the intensive care unit was also longer for group NB at 4 (3–5) days compared to 3 (3–4) days for ESB (p: 0.001). Ejection fraction values, cardiopulmonary bypass, and aortic cross-clamp times were similar in both groups. Postoperative 24 h troponin I levels were higher for group NB at 0.94 (0.22–2.70) mcg/L compared to 0.16 (0.06–1.40) mcg/L for group ESB (p: 0.016)

**Conclusion:**

It would be useful for anesthesiologists to know that erector spinae plane blocks applied in the preoperative period in cardiac surgeries not only shorten the mechanical ventilation and hospitalization times but also provide lower troponin values in the postoperative period patient follow-ups.

## 1. Introduction

Procedures such as skin incision and sternum retraction performed during cardiac surgeries cause moderate to severe pain in the postoperative period [1]. Postoperative pain restricts breathing, impairs secretion excretion, and increases the risk of atelectasis and pulmonary infections. Effective and appropriate postoperative analgesia methods can help prevent complications resulting from surgical trauma. This, in turn, can shorten extubation times, reduce intensive care unit (ICU) stays, and even decrease mortality. There are various methods available for providing postoperative analgesia in open heart surgery, including intravenous use of opioid analgesics, patient-controlled analgesia devices, epidural analgesia techniques, local infiltration methods, paravertebral blocks, nonsteroidal antiinflammatories, and minimally invasive surgical techniques [2].

The erector spinae plane (ESP) block, first described by Forero et al., is a trunk block that can be used for postoperative analgesia. It involves the application of a local anesthetic to the plane between the erector spinae muscle and the transverse process of the thoracic vertebra using ultrasound guidance. The local anesthetic spreads in the craniocaudal and anteroposterior planes, blocking the branches of the spinal nerves at multiple levels. While the sternum is innervated by the nerves between T2-T6, the ribs and surrounding skin are also innervated by the thoracic nerve roots [2,3]. Therefore, there are ESP block studies to prevent postoperative pain in open heart surgeries.

The primary aim of this study is to examine the effects of preoperative bilateral ESP block on postoperative extubation time and laboratory parameters in open heart surgery. The secondary objectives are to compare the postoperative mean arterial pressures, inotropic usage amounts, atrial fibrillation assets, and length of stay in the intensive care unit for patients with and without the block.

## 2. Materials and methods

This study obtained ethical approval from the Clinical Research Ethics Committee of the Medical Faculty of Kahramanmaraş Sütçü İmam University (decision no: 2022/23–04, on 16.11.2022). The records of 320 patients who underwent open-heart surgery (AVR, MVR, CABG) between September 2019 and September 2022 were retrospectively reviewed by the departments of cardiovascular surgery and anesthesia. Data from patients who met the study criteria were recorded retrospectively from the hospital information management system (HIMS) and anesthesia follow-up forms. The included patients underwent cardiac bypass surgery under general anesthesia, were extubated within 24 h postoperatively, and were operated on by the same surgical and anesthesia teams. A total of 235 patients were excluded from the study, including those who underwent off-pump surgery, presented with sepsis, were intubated for the operation, underwent emergency and/or redo surgery, received left ventricular assist device placement, and had advanced liver-kidney failure (AST/ALT > 1.5, INR > 3.5, prothrombin time > 50 s, serum bilirubin > 18 mg/dL, glomerular filtration rate < 30 mL/dk/1.73 m^2^) and inflammatory pathologies (such as rheumatoid arthritis, ankylosing spondylitis). ESP block is routinely performed in our clinic by our anesthesia team. Our procedure involves the bilateral ESP block (details at “2.1. Erector Spinae Plane Block Method” part). The patients included in our study were divided into two groups. Patients who received ESP block were referred to as the ESB group, while those who were nonblock were referred to as the NB group. Demographic data such as age, sex, comorbidities, medications used, surgical procedure, height, weight, and body mass index (BMI) of the patients were recorded. Additionally, ejection fraction (EF) value, duration of cardiopulmonary bypass (CPB) and aortic cross-clamp (ACC), extubation time, and length of stay in the intensive care unit (ICU) were documented from anesthesia and ICU monitoring forms. CKMB and troponin I values at 24 h were recorded. The amount of inotropic support used in the first 24 h, as well as urea, creatinine, urine output, and lactate levels at the 1st, 12th, and 24th h postoperatively, were also recorded. All these data were scanned and recorded from patient follow-up forms and the HIMS. The presence of statistical significance was investigated between the ESB and NB groups regarding extubation time and laboratory parameters.

### 2.1. Erector spinae plane block method

All blocks were performed by the same experienced practitioner in the preoperative waiting room. The patients underwent a standard monitoring procedure (electrocardiography measurements, pulse oximetry monitoring, and noninvasive blood pressure monitoring). After the nasal oxygen cannula was inserted while the patient was in the prone position, sedation was applied with 2 mg midazolam and 25 μg fentanyl. The skin area to be blocked was sterilized using povidone-iodine. A high-frequency linear USG (Logiq™ E7 GE portable ultrasound unit, GE Healthcare, Milwaukee, WI, USA) transducer was placed in a longitudinal position 3 cm laterally at the level of the T5 spinous process. Three muscles were identified: trapezius, rhomboid major, and erector spinae. A 22 gauge, 100 mm block needle (Stimuplex Ultra 360^®^, B. Braun, Melsungen, Germany) was advanced from caudal to cephalic direction using an in plane technique. After contact of the needle with the transverse process, separation was confirmed in the fascial plane by injecting 2 mL of saline after negative aspiration. Then, 20 cc of 0.25% bupivacaine (0.5% plain bupivacaine 10 cc + distilled water 10 cc) was injected with intermittent negative aspirations. The procedure was performed bilaterally.

### 2.2. Anaesthesia management

In our clinic, patients routinely receive midazolam (0.07–0.1 mg kg^−1^), fentanyl (3–5 μg kg^−1^), and rocuronium (0.6 mg kg^−1^) for induction. Anesthesia maintenance is achieved using sevoflurane (0.5–1 MAC). Before the pump, 400 IU kg^−1^ heparin (Vasparin^®^, Vem, İstanbul, Turkiye) was administered intravenously. “Active Clotting Time” (ACT) was kept >400 s. Sevoflurane administration from the CPB machine continued throughout CPB. Intermittent rocuronium and midazolam administration was continued via pump. Once the coronary artery anastomoses were completed, 1 mg protamine sulfate for every 100 IU heparin was administered intravenously for the initial heparin dose. Intermittent arterial blood gas monitoring was performed as standard throughout the operation. The patients were transferred to the cardiac surgery intensive care unit in an intubated state following the standard postoperative analgesia procedure of our clinic, which involved the administration of intravenous morphine at a dose of 0.1 mg kg^−1^ at the end of the case. A standardized protocol for ventilator weaning has been followed for all patients.

### 2.3. Statistical analysis

The recorded data were transferred to the computer with the statistical package program and analyzed with the SPSS 22.0 (IBM Statistical Package for the Social Sciences; Armonk, NY, USA) program. Descriptive statistics were expressed as mean ± SD and median (quartile 25%–quartile 75%). The conformity of the variables to the normal distribution of the data was analyzed using the Shapiro-Wilk test. Independent Samples T test was used for normally distributed variables. Group comparisons of the variables that did not conform to the normal distribution were analyzed using the Mann-Whitney U test. Frequency distributions of categorical variables were analyzed with the Chi-Square test and the Exact test. Statistical significance was accepted as p < 0.05.

## 3. Results

The medical records of 320 patients who underwent open heart surgery were retrospectively analyzed from patient files and HIMS. Among these patients, eighty-five met the study criteria. Two groups were formed: ESB group (n = 40) and NB group (n = 45). The ESB group had 12 female patients (30%), with a mean age of 54.2. The NB group had 16 female patients (35.6%), with a mean age of 55.04. There are no demographic differences among the groups as shown in Table 1. The postoperative extubation time was shorter in the ESB group, with a median of 270 min (240–390), compared to 360 min (300–420) in the NB group as shown in Table 2 (p: 0.006). The intensive care unit stay was longer in the NB group, with a median of 4 days (3–5), compared to 3 days (3–4) in the ESB group as shown in Table 2 (p: 0.001). EF values, platelet counts, CPB and ACC durations were similar in both groups as shown in Table 2.

The CKMB values at 24 h postoperatively were similar in both groups, while the troponin I values at 24 h postoperatively were lower in the ESB group, with a median of 0.16 μg/L (0.06–1.40), compared to 0.94 μg/L (0.22 – 2.70) in the NB group as shown in Table 2 (p: 0.016). The amount of inotropic use, lactate levels, and 1st, 12th, and 24th-h urea, creatinine values, and urine output were similar in both groups as shown in Table 3.

## 4. Discussion

In our study, we found that the application of bilateral ESP block during the preoperative period had an effect on postoperative extubation time, duration of intensive care unit stay, and troponin I value.

Early extubation following cardiac surgery has been associated with increased survival and rapid postoperative recovery. Therefore, reducing the duration of mechanical ventilation results in decreased length of stay in the intensive care unit and hospital [4,5]. It has been reported that ESP block in minimally invasive cardiac surgery shortens the time to extubation and intensive care unit discharge [6]. Similarly, continuous ESP block has been reported to reduce these durations in open heart surgery [7]. However, a meta-analysis by Morgan et al. examining erector spinae plane block in sternotomy cardiac surgeries did not observe a decrease in extubation time in the groups where the block was applied in five studies. Additionally, four studies did not show a decrease in the length of stay in the intensive care unit and hospital [8]. In our study, we obtained results of rapid extubation and rapid intensive care unit discharge in patients undergoing open heart surgery with a single application of bilateral ESP block.

Periprocedural myocardial infarction (PMI) is frequently encountered in cardiac surgeries [9]. PMI progresses with elevation of cardiac enzymes as a result of myocardial damage, and cardiac troponin is an important biomarker in its diagnosis [10]. Zhou et al. reported that high cardiac troponin levels were more powerful in predicting the risk of cardiovascular events, in addition to advanced age (≥65 years), previous percutaneous cardiac interventions, and the number of stents placed [11]. Myocardial injury is a common complication of cardiac surgery and is associated with increased mortality. Postoperative myocardial damage can be assessed by measuring troponin I levels. Cardiac troponin I peak within 30 days postoperatively was associated with an increased risk of death in cardiac surgeries [12]. Surgical stress activates the sympathetic system. Uncontrolled pain also activates the sympathetic system in the postoperative period, impairing coronary perfusion with the effect of tachycardia and hypertension, and reducing myocardial oxygen delivery. Therefore, myocardial infarction is triggered [13]. It is thought that local anesthetic injected into the target interfascial plane in ESP block reaches the sympathetic chain after the paravertebral space and causes sympathetic blockade [14]. It has also been reported that ESP applied during general anesthesia is beneficial in reducing the levels of ACTH, cortisol, and noradrenaline, which are stress response indicators, in patients undergoing thoracoscopic radical resection [15].

In the literature review conducted by Misra et al., it was reported that there was a low increase in postoperative troponin levels in patients undergoing open heart surgery when an ESP block was applied [16]. In the group of patients who underwent ESP block during off-pump coronary artery bypass surgery, lower postoperative troponin concentration was observed [17]. In our study, we observed that troponin levels at the postoperative 24th h were lower in the group with ESP block than in the group without block. We attribute this positive effect to the reduction of sympathetic stimulation and surgical stress response of ESP block. As the rate of oxygen delivered to the myocardium improves, the increase in troponin levels can be prevented.

The technique in the erector spinae plane block involves the application of local anesthetic between the transverse process of the vertebra and the erector spinae muscle [18]. In a cadaver study, it was reported that the contrast agent, representing local anesthesia, exhibited spread beyond the erector spinae muscle to the intercostal spaces, neural foramina, ventral and dorsal branches of thoracic and abdominal nerves, and the epidural space [19]. The idea of blockade of sympathetic fibers by spreading to the epidural and paravertebral spaces supports the visceral analgesia mechanism of ESP block. In addition, the injection site in the ESP block is away from major vascular structures, making it safe to use in cardiac surgery and patients receiving anticoagulant or antiplatelet therapy [20]. Our main goal is to reduce opioid consumption for postoperative analgesia and support rapid recovery and discharge.

It is important to note that our study had limitations due to its retrospective nature and small sample size. To further validate these findings and better understand the myocardial protective effects of ESP blocks and their relationship with troponin values, larger multicenter prospective studies are warranted. In addition, although it has been observed that postoperative pain scores and analgesic consumptions are lower in the ESP block group in practice, another limitation of the study is that we cannot report the effect of ESP on postoperative pain with clear data due to the inability to access the relevant records in retrospective examinations. Despite the limitations, our results suggest that ESP blocks have the potential to improve postoperative outcomes and may serve as an effective analgesic technique in cardiac surgeries.

## 5. Conclusion

In conclusion, our study suggests that preoperative bilateral ESP blocks in open heart surgery can have beneficial effects. The block was associated with shorter postoperative mechanical ventilation duration and hospital length of stay compared to intravenous analgesia. Furthermore, the block appeared to prevent the increase in postoperative troponin values, indicating a potential myocardial protective effect. However, further comprehensive randomized controlled studies are needed to determine the efficacy of ESP blocks in cardiac and other surgical procedures, with the goal of widespread application in the future.

## Figures and Tables

**Table 1:** Comparison of demographic data of patients.

		ESB Group	NB Group	p
**Sex**	Female, n (%)	12 (30%)	16 (35.6%)	0.586
	Male, n (%)	28 (70%)	29 (64.4%)	
**Age (years)**	Mean ± SD	54.20 ± 9.57	55.04 ± 9.34	0.682
**Weight**	Mean ± SD	78.00 ± 11.11	75.62 ± 11.85	0.344
**Height**	Mean ± SD	167.50 ± 6.42	164.84 ± 8.42	0.109
**BMI**	Mean ± SD	27.72 ± 3.77	27.54 ± 4.49	0.845
**Type of surgery**	AVR, n (%)	7 (17.5%)	7 (15.6%)	0.368
	AVR+MVR, n (%)	3 (7.5%)	6 (13.3%)	
	CABG, n (%)	24 (60.0%)	29 (64.4%)	
	MVR, n (%)	6 (15.0%)	2 (4.4%)	
	CABG+AVR, n (%)	0 (0.0%)	1 (2.2%)	
**Additional diseases**	No disease, n (%)	17 (42.5%)	16 (35.6%)	0.552
	HT, n (%)	9 (22.5%)	9 (20.0%)	
	DM, n (%)	5 (12.5%)	6 (13.3%)	
	HT+DM, n (%)	9 (22.5%)	11 (24.4%)	
	Asthma+COPD, n (%)	0 (0.0%)	3 (6.7%)	
**Medications used**	No drug, n (%)	11 (27.5%)	14 (31.1%)	0.130
	OAD/Insulin, n (%)	3 (7.5%)	3 (6.7%)	
	OAHT, n (%)	10 (25.0%)	2 (4.4%)	
	OAD/Insulin +OAHT, n (%)	8 (20.0%)	11 (24.4%)	
	CCB, n (%)	2 (5.0%)	2 (4.4%)	
	BB+ASA, n (%)	6 (15.0%)	13 (28.9%)	

BMI: body mass index, AVR: aortic valve replacement, MVR: mitral valve replacement, CABG: coronary artery bypass graft, HT: hypertension, DM: diabetes mellitus, COPD: chronic obstructive pulmonary disease, OAD: oral antidiabetic, OAHT: oral antihypertensive, CCB: calcium channel blocker, BB: beta blocker.

Independent samples t-test; Chi-Square test; Exact test; a: 0.05.

**Table 2:** Comparison of perioperative features between groups.

	ESB Group	NB Group	p
**E/F (%), median (Q1**–**Q3)**	60.00 (55.00–60.00)	55.00 (50.00–60.00)	0.144
**CPB time (min), median (Q1**–**Q3)**	97.50 (81.00–119.50)	95.00 (75.00–112.00)	0.517
**ACC time (min), median (Q1**–**Q3)**	57.50 (46.50–79.00)	56.00 (46.00–73.00)	0.721
**PO ext time (min), median (Q1**–**Q3)**	270.00 (240.00–390.00)	360.00 (300.00–420.00)	**0.006** *
**ICU length of stay (day), median (Q1** –**Q3)**	3.00 (3.00–4.00)	4.00 (3.00–5.00)	**0.001** *
**PO 24th h CKMB (U/L), median (Q1** –**Q3)**	9.30 (2.30–27.00)	7.60 (2.60–15.90)	0.774
**PO 24th h troponin I (mcg/L), median (Q1**–**Q3)**	0.16 (0.06–1.40)	0.94 (0.22–2.70)	**0.016** *

ACC: aortic cross-clamp, CPB: cardiopulmonary bypass, E/F: ejection fraction, Ext: extubation, ICU: intensive care unit, PO: postoperative.

Mann Whitney U test; a: 0.05;

* Statistical significance.

**Table 3:** Comparison of clinical and laboratory parameters between groups.

	ESB Group	NB Group	p
**PR urea (mg/dL), median (Q1**–**Q3)**	16.0 (13.0–21.0)	15.0 (13.0–18.0)	0.446
**PO 1st h urea (mg/dL), median (Q1**–**Q3)**	18.00 (15.00–22.00)	15.50 (13.25–19.50)	0.064
**PO 12th h urea (mg/dL), median (Q1**–**Q3)**	18.00 (14.00–23.00)	18.00 (14.00–22.00)	0.605
**PO 24th h urea (mg/dL), median (Q1**– **Q3)**	19.00 (14.00–24.00)	20.00 (16.00–28.00)	0.392
**PR cr (mg/dL), median (Q1**–**Q3)**	0.86 (0.75–1.01)	0.80 (0.64–0.98)	0.147
**PO 1st h cr (mg/dL), median (Q1**–**Q3)**	0.87 (0.74–1.01)	0.83 (0.65–0.98)	0.285
**PO 12th h cr (mg/dL), median (Q1**–**Q3)**	0.85 (0.70–0.98)	0.87 (0.72–1.03)	0.837
**PO 24th h cr (mg/dL), median (Q1**–**Q3)**	0.82 (0.69–0.94)	0.91 (0.68–1.07)	0.360
**PO 1st h urine (mL), median (Q1**–**Q3)**	375.00 (250.00–500.00)	500.00 (375.00–500.00)	0.108
**PO 12th h urine (mL), mean ± SD**	2063.24 ± 843.27	2331.59 ± 801.96	0.156
**PO 24th h urine (mL), mean ± SD**	2993 ± 902	3467 ± 898	**0.024** *
**PO 1st h lac (mmol/L), median (Q1**–**Q3)**	2.00 (1.00–3.40)	2.20 (1.50–3.20)	0.331
**PO 12th h lac (mmol/L), median (Q1**–**Q3)**	2.20 (1.40–3.40)	1.80 (1.30–3.20)	0.649
**PO 24th h lac (mmol/L), median (Q1**–**Q3)**	2.30 (1.60–3.10)	1.90 (1.20–2.60)	0.147
**PO dopamine (mg), median (Q1**–**Q3)**	30.00 (9.00–44.00)	36.00 (17.00–62.00)	0.178
**PO norepinephrine (mg), median (Q1**–**Q3)**	1.57 (0.00–24.00)	4.50 (0.00–13.00)	0.921
**PO dobutamine (mg), median (Q1**–**Q3)**	0.00 (0.00–0.00)	0.00 (0.00–0.00)	0.509
**PO adrenaline (mg), median (Q1**–**Q3)**	0.00 (0.00–0.00)	0.00 (0.00–0.00)	0.392

Cr: creatinine, Lac: lactate, PR: preoperative, PO: postoperative, H: hour.

Independent samples t-test; Mann Whitney U test; a: 0.05;

* Statistical significance.
